# Statistics on the burden of dementia: need for stronger data

**DOI:** 10.1016/S1474-4422(18)30456-3

**Published:** 2018-11-26

**Authors:** Lenore J Launer

**Affiliations:** Neuroepidemiology Section, Intramural Research Program, National Institute on Aging, National Institutes of Health, Bethesda, MD 20892, USA

Dementia primarily affects an individual’s cognitive function and many aspects of life are negatively affected by cognitive decline. There are no approved disease-modifying drugs and no approved prevention strategies for dementia; a heavy burden is placed on the individual who has dementia, their family, and society. In *The Lancet Neurology*, a report^1^ from the Global Burden of Diseases, Injuries, and Risk Factors Study (GBD) 2016 Dementia Collaborators presents estimates of dementia-related deaths, prevalence, quality of life measures, and risk factors, with the aim of documenting global patterns and providing data for research, and to guide a wide range of public health investments.

Calculations were based on the GBD models that have been used to estimate the burden of more than 300 diseases and injuries in 195 countries and territories.^2^ Because of the marked inconsistencies in the location-specific data for prevalence and incidence of dementia and mortality, and the marked heterogeneity in the studies included in this report, several of the assumptions that are usually used in the GBD methods could not be met. Therefore, the type of source data used and the modelling approaches were modified so that the data fitted the assumptions of the core GBD models. For example, for locations that did not have data available it appeared that the ratio of prevalence to cause-specific mortality from the USA, Puerto Rico, Finland, and Sweden were incorporated to estimate cause of death, prevalence, quality of life, and risk factors for dementia.

The report^1^ makes an important point about the huge burden of dementia: in 2016, the global number of individuals who lived with dementia was 43·8 million (95% uncertainty interval [UI] 37·8–51·0), increased from 20·2 million (17·4–23·5) in 1990. The report also provides an opportunity to examine further the way in which dementia statistics are generated, particularly because obtaining reliable and valid dementia case counts presents challenges that do not apply to obtaining such data for many other common diseases.

Dementia is a multisystem condition. The definition is still evolving in research and clinical communities, and this affects how data are entered into administrative databases. Case ascertainment depends upon several aspects, such as the health-care infrastructure of a community, socioeconomic conditions, cultural norms, access to health care, age structure, modes of caring for elderly people, and recognition of pathological changes in function. Despite efforts to standardise dementia and Alzheimer’s disease assessments since the early 1980s, dementia is still assessed in many different ways. Mild cases are not reliably identified, the terminology and categorisation of dementia have shifted over time, and there are differences in regional medical society guidelines, local context, and resources. The diagnosis can be operationalised by clinical judgment, algorithms, or questionnaire scales. There has also been a proposal^3^ to define Alzheimer’s disease, typically the most common clinically defined subtype of dementia, by biomarker criteria derived from MRI scans and from PET scans or CSF, regardless of cognitive function. If this proposal moves beyond a narrow research framework and into broader research efforts and clinics, further complexities will be created in data interpretation and use. For example, such criteria might result in an increased number of people diagnosed with dementia who have no functional impairment, or in fewer cases being identified in regions with no means to gather data on the proposed markers.

Going forward, there might also be challenges to our underlying models of prevalence and life lived with dementia. The currently accepted model is an exponential age-related increase in prevalence and incidence of dementia, with few cases occurring before age 70 years and many, but a poorly estimated number of, cases after age 90 years.^4,5^ However, emerging data suggest that this model might be changing. For example, a study of data based on a large medical records database suggested there might be an increase in an alcohol-related earlier onset dementia;^6^ several epidemiological studies have suggested there is a decline in incident cases of dementia;^7^ people with dementia who are aged 90 years or older have different presentations compared with those who develop dementia at an earlier age as their presentation is complicated by multiple-morbidity;^8^ and improvements in treating chronic diseases extend life, and therefore an increase in the number of people living with dementia is expected. At the same time, the increase in the occurrence of risk factors, such as diabetes,^5^ at younger ages means that individuals might be exposed to risk sooner and possibly have an earlier onset of signs of dementia.

Although collecting data for public health purposes has different aims from those of research into the causes of dementia,^4^ both are needed to reduce the burden of disease. The GBD 2016 Dementia Collaborators^1^ report that 6·4 million (95% UI 3·4–10·5; 22·3%) of the total DALYs caused by dementia could be attributed to four modifiable risk factors that met GBD criteria for analysis (high body-mass index, high fasting plasma glucose, smoking, and a diet high in sugar-sweetened beverages). To put these findings into context, several reviews of risk factors^5^ and methods to study dementia^9^ are helpful. These sources have pointed out the complexities of understanding the trajectories of cognitive decline and risk factors, and the difficulties in interpreting studies that do not take into account the limitations of the study design and issues such as the selective loss over time of sicker individuals from the study, the quality and appropriateness of exposure and outcome measures, and the choice of statistical models.

From a public health and disease-prevention perspective, too few quality data are available for dementia that fit the complex reality of this devastating public health problem. Additionally, it is questionable whether the extant data are strong enough to help achieve the goals of this GBD study—to inform policy makers, researchers, and clinicians about global differences in dementia trends, clusters of dementia, and causal risk factors. To reach these goals, several areas of data collection and interpretation require strengthening: improvement of research methods used in data collection and interpretation; development of a consensus about valid coding of dementia for administrative databases; and development of flexible approaches that take into account the variation in place and over time of health and social conditions that might lead to severe cognitive impairment.
